# Exergaming in augmented reality is tailor-made for aerobic training and enjoyment among healthy young adults

**DOI:** 10.3389/fpubh.2024.1307382

**Published:** 2024-02-26

**Authors:** Antony G. Philippe, Aurélie Goncalves, Karim Korchi, Maxime Deshayes

**Affiliations:** UNIV. NIMES, APSY-V, Nîmes, France

**Keywords:** physical activity, enjoyment, accelerometry, heart rate, exergaming, augmented reality, endurance training, health promotion

## Abstract

In recent years, the use of exergaming for physical activity practice has gain in popularity but few is known about the use of augmented reality for physical activity, particularly at moderate to vigorous intensities. The present study examined the use of an exergame in augmented reality for aerobic training in healthy young adults. In a within-subject design, 18 participants (19.8 ± 1.4 years of age) have performed two physical activity sessions playing dodgeball. Indeed, they realized a classical dodgeball session and an exergaming session with an augmented reality version of dodgeball game. Physical loads and intensities were measured with accelerometers, RPE and heart sensors. Enjoyment experienced during the sessions was measured with the short version of the physical activity enjoyment scale questionnaire. Results revealed that both physical load and intensity were appropriate for aerobic training in the two conditions (i.e., classical and exergame in augmented reality) although values were significantly higher in the classical condition. Enjoyment was high in the two conditions with a higher significant value in the classical condition compared to the exergame in augmented reality condition. Put together, these results indicate that an aerobic state can be attained through both physical gameplay and its augmented reality equivalent and was associated to a high level of enjoyment among healthy young adults.

## 1 Introduction

Sedentary behavior and physical inactivity have a negative impact on health. It is known to increase physiological risks such as heart disease, high blood pressure, and Type 2 diabetes (1, 2). Also, it can lead to psychological issues such as depression, anxiety, and low self-esteem (3). These troubles and risk factors can be associated with weight gain, obesity, risk of falls, coordination impairment, sarcopenia, and osteoporosis (4–6). Thus, physical activity (PA) associated with a healthy lifestyle appears to be important to limit these deleterious effects on health. The promotion of active behavior is thus essential to prevent health deterioration among life-course. Despite recommendations from the World Health Organization (WHO) regarding PA (7), sedentary behavior is increasingly prevalent (8, 9), and there is a significant decline in PA among adolescents and college students as they age (9, 10). Worldwide, only 20% of adolescents attain 60 min of moderate-to-vigorous physical activity (MVPA) per day (8). Entering university is generally associated with a decrease in PA practice and an increase in sedentary behavior (11) and college students have generally a low level of engagement in PA (12). Nonetheless, regular PA can improve physical fitness among college students and reduce the risk of developing chronic diseases (13, 14) appears to be a protective factor against stress and mood disorders (3).

The decrease in global PA level can be associated to the rise of digital technology. Indeed, with easy access to the Internet on mobile devices, screen time has progressively replaced physically active behaviors (15). Instead of reducing the use of technology, novel strategies are to wisely use it to promote PA and active behaviors (16). For example, exergaming programs can be used to increase health and exercise behaviors in adults (17). Exergaming has been associated with benefits on both physiological (18) and neurological (19) outcomes when performed at a sufficient intensity (17) and can be used to increase the weekly dose of PA for health benefits. Numerous studies have shown that the higher enjoyment during PA, the higher PA loads and engagement (20–22), which is in line with the self-determination theory (23). This suggests that enjoyment, which is inherently laden with psychosocial, physiological, and embodiment substrates, is a lever to PA practice and active behaviors. Moreover, enjoyment has been linked to participation in games (24) and exergaming (17, 18). With exergames, it has been shown that enhancing the fun or enjoyment experienced during the games could enhance the intensity and duration of PA, and thus, the health benefits (24). Taken together, these data suggest that exergaming appears to be an efficient approach to promoting PA.

Exergaming is in perpetual evolution. In recent years, new technologies such as virtual reality (VR) and augmented reality (AR) have been developed. Indeed, in AR, 3D virtual objects are integrated into a 3D real environment in real-time (25). AR became a standard household technology with the release of the game Pokémon GO in 2016 (26). Nowadays, AR is mainly used in the domain of health, in the management of gait impairment among the older adults, or medicine and surgery (26–30). For the promotion and practice of PA in healthy adults, Pokémon GO remains the main use of AR. Nevertheless, Pokémon Go is associated with a transient increase in PA load during the first week and low PA intensity in healthy young adults with better effects of PA levels among participants with a lower baseline of PA level and those who were overweight/obese (31, 32). Data is lacking concerning the use of AR for aerobic training in healthy and physically active young adults. To the best of our knowledge, the use of exergaming in AR has never been associated with a high-intensity PA nor has been used for moderate to high intensity training in healthy young adults.

Among team sports practiced at moderate to vigorous intensities, dodgeball has become increasingly popular among children and adults (33). In this team sport played by two teams, players throw balls and try to hit their opponents while avoiding being hit themselves. The popularity of this sport has led to the development of an AR version named HADO. In HADO, players wear an AR headset and a connected bracelet to throw virtual energy balls instead of real balls. Nevertheless, little is known about the physiological effects of this exergame and on enjoyment experienced during an exergame session in AR. Thus, the present study aimed to (i) test whether PA load and intensity during an exergame session in AR (HADO) were sufficient for aerobic training and (ii) check if enjoyment experienced during this session was high enough to use AR for the promotion of active behaviors among healthy college students.

## 2 Method

### 2.1 Participants and study design

Eighteen subjects participated in the study. Subjects were 18–23 years of age (19.8 ± 1.4 years old) including five women. Participants were students recruited from the University of Nîmes, France. All participants provided informed consent and a valid medical certificate allowing the practice of PA. Exclusion criteria included physical diseases that prevent PA practice (e.g., cardiovascular disease), and visual disturbance. According to the Ricci and Gagnon questionnaire (34), participants were considered physically active (scores equal to 28 ± 5; with active defined as a total score ≥ 18) (34, 35). The resting heart rate (HR_r_) was 70.3 ± 12.4 bpm.

The study employed a within-subject design because paired samples tests (*t*-test and Wilcoxon test) are used to determine whether the change in means between two paired observations is statistically significant (36). In this test, same subjects are measured in two different conditions. This design has been used because (i) it has greater statistical power (37) and (ii) it allows better observation of individual differences, as each participant is assigned to each condition (i.e., exergaming in AR session and classical dodgeball session).

### 2.2 Physical activity intervention

Participants were divided into groups of 6 people who had to participate in 3 1-h PA sessions spread over three consecutive weeks, in the afternoon. All of them began with a familiarization session including exergaming in AR and classical Dodgeball playing, followed by two randomized experimental conditions: AR dodgeball condition (AR session) and classical dodgeball condition (Classica PA session). All sessions were performed in the same dance hall, on a 10 × 6 meters delimited space. Indeed, all sessions were spread on 9 weeks: 3 consecutive weeks × 3 for the 3 groups of 6 participants.

#### 2.2.1 Familiarization session

The first session was focused on familiarization with the tools and expectations. Objectives were to get used with the equipment including accelerometers, heart sensor, and the HADO display (HADO^®^, France). During this session, participants learned the rules of dodgeball and HADO and how to equip and use the HADO display. Since PA was monitored during the two other sessions (heart sensor and accelerometers), participants needed to be at ease with the session content and equipment to not misestimate PA load. This session started with a 10-min general warm-up (footing, jump, burpees, etc.) followed by 20 min of classical dodgeball and 20 min of exergame in AR with HADO.

#### 2.2.2 Classical dodgeball session

**Figure 1:**
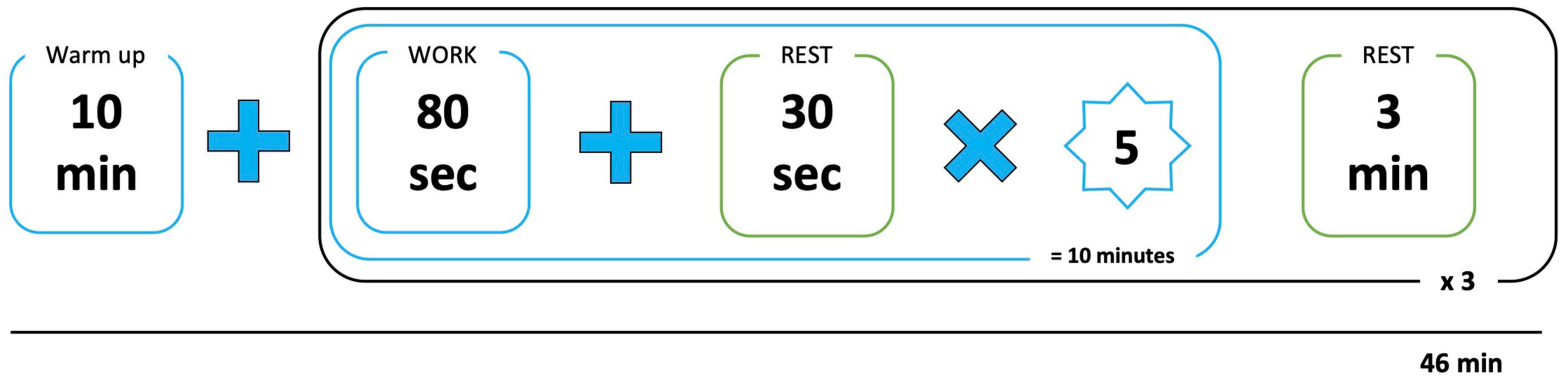
Experimental sessions content.

Classical sessions included a 10-min warm-up, similar to the familiarization session, followed by 36 min of dodgeball. Dodgeball was played at 3 vs. 3 players, in socks, with 6 foam balloons. The session’s content is fully described in Figure 1.

#### 2.2.3 Exergaming in AR session

**Figure 2:**
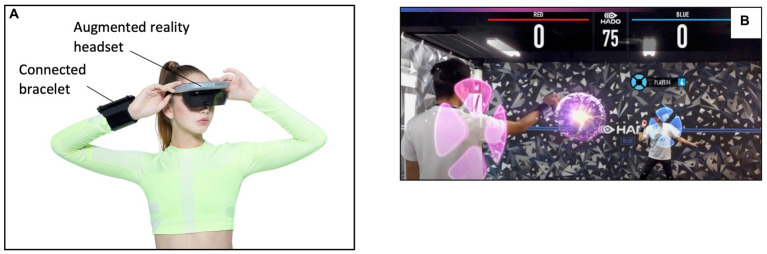
HADO equipment **(A)** and in game view **(B)**.

AR sessions included a 10-min warm-up, similar to the familiarization session, followed by 36 min of HADO exergame in AR (description in Figure 1). HADO is a next-generation dodgeball game in which two teams of 3 players confront each other by sending virtual energy balls to score points by hitting the opponents. Players wear an AR headset designed especially for the HADO game and a connected bracelet that captures upper limb movement to perform various actions such as throwing an energy ball and reloading (Figure 2A). The headset includes an iPhone 8 (Apple Inc., Cupertino, CA, United States) with a retina screen of 4.7 inches, a resolution of 1,334 × 750 pixels and a frame rate of 60 Hz. The total weight of the headset was 248 g. The connected bracelet includes an Ipod touch 7th (Apple Inc., Cupertino, CA, United States). An example of what players see during the game is presented in Figure 2B.

### 2.3 Materials and measures

#### 2.3.1 Physical activity load and intensity measurements

PA load was measured objectively and subjectively during each PA session.

Subjective PA load was evaluated according to the rate of perceived exertion (RPE). After the session, participants had to report the perceived intensity of the session on a CR-10 RPE scale (38). This method has previously been used to monitor PA load in various physical activities (39–41). See Haddad and collaborators for review (42).

Objective PA load was measured with tri-axis accelerometers GT3X (Actigraph, Pensacola, FL, United States). Participants had to wear the accelerometer on the right side of the hip, adjusted with an elastic belt, during the three PA sessions (43). Actilife v-6.13.4 Lite Pro software was used to extract PA values. Data were downloaded in a 1-s epoch to measure PA. According to the Freedson algorithm, we defined MVPA as >1,952 counts per minute (44). MVPA was measured during the session.

Heart parameters, i.e., resting HR (HR_rest_), maximal HR during the PA sessions (HR_max_) and mean HR during the PA sessions (HR_mean_) were examined as physiological parameters. Polar H10 sensor was used. This sensor is attached to a chest strap and placed on the xiphoid process of the sternum with the chest strap fitted around the participant’s chest (45). This method has previously been validated against the electrocardiogram goal standard (46). Data were collected with the Polar Beat app installed on Samsung Galaxy Tab S6. HR was measured during the 46 min of the effective time of PA of the 1-h total session. HR_rest_ was measured in the sitting position, after 10 min of rest during the familiarization session. Moreover, theoretical maximal HR (HR_maxT_) was estimated with the Fox-HR_max_ equation (220-age) that has been previously validated in healthy adults (47–49).

Intensity of PA sessions was expressed in percentage of maximal intensity and calculated as follows:


$$\mathrm{Intensity}\;\left(\%\right)=\frac{{HR}_{\mathrm{mean}}}{{HR}_{\mathrm{maxT}}}*100.$$

Thus, during the entirety of each session, participants had to wear a heart rate sensor and an accelerometer. To reduce variability, participants were given the same accelerometer and heart rate sensor for the three sessions and all sessions were conducted by the same experimenter.

#### 2.3.2 Enjoyment

To measure enjoyment, participants had to complete the short version of the physical activity enjoyment scale (PACES-S) at the end of each PA session. This short version of the original PACES (50) has recently been validated by Chen and collaborators (51). PACES-S consists of 4 items based on a Likert scale scored from 1 (strongly disagree) to 5 (strongly agree) with a total maximal score of 20.

### 2.4 Analysis

Variable data normality was checked using the Shapiro–Wilk test and homogeneity of variance with the *F*-test. When normality was assumed, the parametric paired-sample *t*-test was performed. When study variables displayed a deviation from normality, effects were analyzed by the non-parametric paired-sample Wilcoxon test. Analyses were performed using JASP software (version 0.14.1, JASP Team). Data are presented as means ± SD. Significance was set at *p* < 0.05.

## 3 Results

Means and SDs for the study variables are presented in Table 1.

**Table 1:** Means (± standard deviation) for the studied variables.

	PA session in AR	Classical PA session	
Variables	Mean (SD)		*p* value
RPE	3.7 (1.2)	5.1 (3.7)	0.02
MVPA	20.6 (3.5)	24.9 (3.6)	0.001
HR_mean_	132.7 (21.4)***	144.2 (12.6)***	0.012
HR_max_	177.4 (21.2)***	187.0 (10.9)***	0.043
Intensity	66.3% (10.9)	72.0% (6.2)	0.012
Enjoyment	17.5 (2.5)	18.6 (1.9)	0.028

PA, physical activity; AR, augmented reality; RPE, rating of perceived exertion; MVPA, moderation to vigorous physical activity; HR_mean_, mean heart rate during the session in bpm; HR_max_, maximal heart rate during the session in bpm. SD, standard deviation; ***Different from HR_rest_ (*p* < 0.001). *n* = 18 for all the studied variables.

### 3.1 Descriptive analysis

#### 3.1.1 Classical PA session

For the classical PA session, the mean subjective PA load was 5.1 ± 3.7 (i.e., RPE on the CR-10 scale). The mean objective PA (i.e., MVPA) was 24.9 ± 3.6 min representing 54% of the effective time of PA.

HR_max_ was 187.0 ± 10.9 bpm and HR_mean_ was 144.2 ± 12.6 bpm. Intensity was 72.0 ± 6.2%. Both HR_max_ and HR_mean_ were significantly different from HR_rest_ (*p* < 0.001).

For the classical PA session, mean enjoyment was 18.6 ± 1.9.

#### 3.1.2 PA session in augmented reality

For the PA session in AR, the mean subjective PA load was 3.7 ± 1.2 (i.e., RPE on the CR-10 scale). The mean objective PA (i.e., MVPA) was 20.6 ± 3.5 min representing 45% of the effective time of PA.

HR_max_ was 177.4 ± 21.2 bpm and HR_mean_ was 132.7 ± 21.4 bpm. Intensity was 66.3 ± 10.9%. Both HR_max_ and HR_mean_ were significantly different from HR_rest_ (*p* < 0.001).

For the virtual PA session, mean enjoyment was 17.5 ± 2.5.

### 3.2 Comparison between classical and augmented reality PA sessions

The paired-sample analysis (Classical PA session vs. augmented reality PA session) revealed a statistical difference between all variables. Indeed, PA load (i.e., RPE, MVPA, HR_mean_, HR_max,_ and intensity) and enjoyment were lower in augmented reality PA sessions compared to classical PA sessions (all *p* < 0.05).

## 4 Discussion

In the present study, PA load, intensity, RPE, and enjoyment of healthy young adults were monitored while they practiced the same PA session under two different conditions (i.e., Classical and AR sessions). Results indicate that (i) PA loads and intensity in both conditions (i.e., Classical and AR dodgeball sessions) were appropriate for aerobic training and (ii) the two conditions were highly appreciated by the participants. Nevertheless, physical loads, intensity and enjoyment were lower in the AR condition and the results are worthy of discussion.

PA intensities (% on HR_maxT_) were 66.3 ± 10.9 and 72.0 ± 6.2% for Classical and AR sessions, respectively. These intensities both correspond to the appropriate range of relative exercise intensity for aerobic conditioning comprised between 60 and 90% of maximal HR (52). This indicates that in our conditions, although intensity was lower in the AR session compared to the Classical session, the use of virtual tools is appropriate for aerobic training. Moreover, for the two conditions, MVPAs were similar to those measured in a previous study in which participants were asked to perform as much effort as possible during the same duration session (45–50 min) (40). This suggests that the PA loads in the present study were close to the maximal load participants could perform for the two conditions (i.e., Classical and AR dodgeball sessions).

As mentioned above, the results indicate that exergame in AR can be used for aerobic training in healthy college students. Nevertheless, another barrier among this population is that they have a low level of engagement in regular PA (12, 53) and do not reach the WHO recommendations in terms of PA (54). A part of the solution is to increase motivation and enjoyment since it has been shown that a high enjoyment score is correlated to higher PA practice (55). This is in line with our result since we have measured a high enjoyment (17.5 ± 2.5 for a maximal score of 20 for the PA session in AR) and that the use of numerical support during the practice of PA can enhance enjoyment and engagement in a regular practice of PA, especially among college students (56, 57). Given that in France, only 71% of men and 53 of women met the PA recommendations (58), our results suggest that AR could be a lever to PA practice among healthy college students and a complementary approach to meet PA recommendations.

To the best of our knowledge, this study is the first to measure and compare PA load, intensity and enjoyment during two sessions, exergame in AR vs. classical sessions, in very similar conditions among healthy adults. In our conditions, the total session duration and effective PA time were similar. Moreover, the activity itself was the same. Participants played dodgeball in both conditions, on the same field with the same experimenter. Previous studies have compared enjoyment and PA loads and intensities but in different conditions. Indeed, studies have compared different immersive VR games (59, 60), but the results were not compared to a similar classical PA. For example, McDonough and collaborators compared traditional treadmill exercises to Just Dance or Reflex Ridge games, which are different in terms of movements and objectives (56). Another study has compared RPE and PA load between traditional exercise bikes and VR-based exercise bikes (61). Results indicate that PA load was higher in de VR-based exercise bike condition compared to the traditional condition. However, PA load was computed as pedal revolution count that is the pedaling speed rather than a physiological parameter. Moreover, all the researches mentioned above have studied the effects of VR which is different from AR. Indeed, AR is used most of the time in surgery or health (62, 63). One of the only contexts where AR is used in healthy people to practice PA is Pokémon GO. Nevertheless, studies indicate that playing Pokémon GO was associated to transient increase in PA level and low intensity PA (31). Thus, Pokémon GO is not suitable to perform PA at moderate or vigorous intensities but can be adapted to populations attracted to the game, that have a lower baseline PA (32).

The present study is the first one to suggest that exergame in AR could be suitable for performing PA at moderate to vigorous intensities. Nevertheless, subjective and objective PA loads (i.e., RPE and MVPA, respectively) as well as heart parameters (i.e., HR_max_ and HR_mean_) and intensities were lower in the AR condition compared to the classical condition. This can be explained by two main reasons. On the first hand, the participants were considered physically active and highly enjoyed the activity. This could explain the higher values observed in the classical conditions because they were not afraid to take risks (run, jump, etc.) and were very engaged in the game. On the other hand, during the AR session, the participants had to wear a head-mounted display. One could say that they were afraid to break it during a jump or a fall during the game. It would have been interesting to measure these fears (fear of getting hurt and fear of breaking the digital equipment). Nevertheless, the relation between risk-taking and the level of PA practice is not known. However, it has previously been shown that motor comportment (i.e., movement) was dependent on the visual display (64). To better understand the relations between engagement in the practice of PA (PA loads and intensity performed), enjoyment, and the initial level of the participant’s PA, it would have been interesting to add a sedentary group. In this way, the comparison of all our variables between active vs. sedentary participants could have provided some complementary answers. Further investigations are needed to explain the differences observed in the present study.

Although PA loads, heart parameters, intensities and enjoyment are lower in the AR condition compared to the classical condition, the use of AR has health benefit. The practice of PA at different intensities and loads can prevent potential injuries due to excessive PA loads (65). Indeed, in endurance training, novice runners have higher risks of injuries than more experienced runners (66, 67). This can be explained by an excessive increment in PA loads (65). This is in line with the strategies of training periodization according to which PA loads variations are recommended (i.e., overloads and tapers) (68). Thus, in the present study, alternating different intensities and PA loads by playing dodgeball in both classical and AR conditions may prevent risks of injuries.

Finally, incorporating motivation as a metric would have been of interest. In healthy young adults, it has been shown that motivational factors in the practice of exergames were associated to the game challenge and the in-game reward system (69). This suggests that incorporating AR in PA could maintain motivation and thus, active behaviors. To confirm this hypothesis, further investigations are needed.

To conclude, this study is the first to highlight that AR (i) is suitable for performing aerobic training among healthy young adults and (ii) is associated with high enjoyment.

## Data availability statement

The raw data supporting the conclusions of this article will be made available by the authors, without undue reservation.

## Ethics statement

Ethical approval was not required for the studies involving humans because Ethical review and approval were not required for the study on human participants in accordance with the local legislation and institutional requirements. Participate to the study had no risks for the health of participants (physiological nor psychological outcomes) and collected data are not considered to be sensitive. Moreover, all data have been anonymized for the analysis and the writing. The studies were conducted in accordance with the local legislation and institutional requirements. The participants provided their written informed consent to participate in this study.

## Author contributions

AP: Conceptualization, Data curation, Formal analysis, Funding acquisition, Investigation, Methodology, Supervision, Writing – original draft, Writing – review & editing. AG: Methodology, Resources, Writing – original draft. KK: Investigation, Software. MD: Conceptualization, Methodology, Writing – original draft, Writing – review & editing.
